# Influence of the osteosynthesis plate on ultrasound propagation in the bone

**DOI:** 10.1590/1413-78522014220500733

**Published:** 2014

**Authors:** Márcio Takey Bezuti, Luiz Garcia Mandarano-Filho, Giuliano Barbieri, Nilton Mazzer, Cláudio Henrique Barbieri

**Affiliations:** 1 Universidade de São Paulo, Faculdade de Medicina de Ribeirão Preto, Ribeirão Preto, SP, Brazil, Faculdade de Medicina de Ribeirão Preto, Universidade de São Paulo, Ribeirão Preto, SP, Brazil

**Keywords:** Acoustics, Ultrasonics, Bone and bones, Bone plates, Steel

## Abstract

**Objective:**

To analyze the influence of steel plates for osteosynthesis on the velocity of ultrasound propagation (VU) through the bone.

**Methods:**

The transverse coronal and sagittal velocity of ultrasound propagation underwater were measured on the intact bone and then on assemblies of the same bone with two types of osteosynthesis plates (DCP and semi tubular), fixed onto the dorsal side of the bones. The first arriving signal (FAS) was the ultrasound parameter used, taking the coronal and sagittal diameters as the distances to calculate velocity. Intergroup statistical comparisons were made at significance level of 1% (p<0.01).

**Results:**

Velocity was higher on the intact bones than on the bone-plate assemblies and higher for the semitubular than for the compression plates, although differences were not statistically significant for most comparisons (p=0.0132 to 0.9884), indicating that the steel plates do not interfere significantly with ultrasound wave propagation through the bone-plate assemblies.

**Conclusion:**

The velocity reduction effect was attributed to the greater reflection coefficient of the steel as compared to that of bone and water. Ultrasonometry can, thus, be used in the evaluation of healing of fractures fixed with steel plates.* Experimental Study.*

## INTRODUCTION

Transmission ultrasonometry has been studied as an ancillary method for the diagnosis of the healing status of fractures by means of experimental, clinical and laboratory bench investigations.^1^^-^ 6 It basically consists of measuring the ultrasound velocity and attenuation, which are fundamental properties of the bone that vary according to structure, density, elasticity and other biomechanical properties, thus supplying an indirect measure of these properties.^7^^-^ 9 It is well established that ultrasound propagation velocity (UV) consistently increases with the healing of a fracture, while attenuation consistently decreases, with both parameters slowly approaching normal values during the remodeling phase. This behavior has a potential for clinical application for the diagnosis of the healing status of a fracture and its anomalies.^10^^,^ 11

Almost all previous investigations about the ultrasonometric diagnosis of the healing status have been conducted on fractures treated conservatively^4^^,^ 11 or with laboratory (phantoms) or computer models without the interference of any metal implant.^12^^,^ 13 However, due to a number of advantages, the conservative methods for the treatment of fractures are progressively being left aside in favor of modern surgical methods. In fact, the use of metal implants for fixing fractures has increased geometrically over the last decades, following the development of modern versions of plates, screws and other devices. As a consequence, internal fixation of fractures has become the gold-standard for the treatment of many fractures, particularly of the diaphysis of long bones, with the predominant use of conventional compression plates due to their versatility and relative low cost.^14^^-^ 16

On the other hand, the universally increasing number of operated fractures also implies an increasing number of complications, including healing anomalies, often with diagnostic problems due to the presence of the metal implant. Actually, plates, screws and intramedullary rods can make it difficult to visualize the fracture itself and occasional healing anomalies. From a theoretical standpoint, a metal implant can interfere with the ultrasound conductivity through the bone, thus potentially altering ultrasound velocity, similarly to anatomical accidents or bone consistency. This hypothesis has not yet been adequately tested after the pioneering study by Saulgozis and collaborators,^10^ who presented the problem but did not deepen the analysis.

Therefore, it was the purpose of the present investigation to analyze the influence of dynamic compression (DCP) and 1/3 semitubular (ST) stainless steel osteosynthesis plates in a model of fresh-frozen sheep tibiae, by means of underwater transverse ultrasonometry according to the coronal and sagittal planes.

## MATERIALS AND METHODS

**Figure 1:**
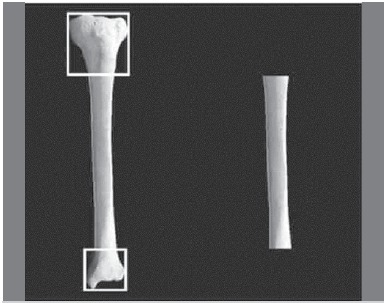
Obtaining the diaphyseal bone segments. The application of Heim´s square (left) and the diaphyseal segment (right).

The investigation was approved by the Ethics Committee on Experimental Use of Animals of our institution. Ten fresh-frozen intact left tibiae of adult sheep (10 months of age, 37 kg body weight on average) were used. For economic and ethical

reasons, the tibiae were taken from animals used in a separate research project, in which the right tibiae had been used. The bones were completely freed from any soft tissue, including the periosteum, by careful dissection and stripping. Both proximal and distal epiphyses were resected by osteotomy at the level of the epiphysis-diaphysis transition, thus leaving just the diaphyseal segment of the bone. To ensure greater homogeneity, the epiphysis-diaphysis transition was located by the application of Heim's square,^17^ designed with all sides equal to the width of the corresponding epiphysis, with the proximal side tangential to the joint line and the opposite side marking the transition. (Figure 1)

At this stage, the volumetric density of the diaphyseal segments was measured to be later applied to the calculation of the acoustic impedance and reflection coefficient of the water-plate, plate-bone and water-bone interfaces. Acoustic impedance (Z) is the product of density (ρ, measured in kg/m^3^) and ultrasound speed (*v*, as measured in m/s), as follows:



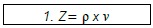



The reflection coefficient corresponds to the amount of ultrasound waves reflected by an interface between two materials for a normal (90º) ultrasound emission and is calculated by the equation:



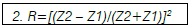



Where R is the reflection coefficient and Z1 and Z2 are the acoustic impedances for the first and second material crossed by the ultrasound waves, respectively. The reflection coefficient varies from 0 to 1 and the value obtained with the above mentioned formula multiplied by 100 yields the amount of energy reflected as a percentage of the emitted energy. The remaining value (1 *minus* R) represents the amount of energy which goes through the interface.

### Fixation technique

The diaphyseal segments were assembled with two types of 8-hole 97 mm-long stainless steel osteosynthesis plates (Synthes Brasil^(r)^, Rio Claro SP, Brazil): 3.5 mm DCP (3 mm-thick) and 3.5 mm 1/3 ST (0.8 mm-tick) plates, according to grouping. Both plate types present the same distance between holes, which permitted using the same bone segments for the entire experiment, changing the assemblies by simply changing the plates. The plates were fixed onto the more even and flatter dorsal aspect of the bone, being adapted so that the respective midpoints coincided, both lengthwise and sideways. For practical purposes, the bone segments were first assembled with the DCP plates, which were replaced with the ST plates, following the corresponding ultrasonometric analysis of each group.

**Figure 2:**
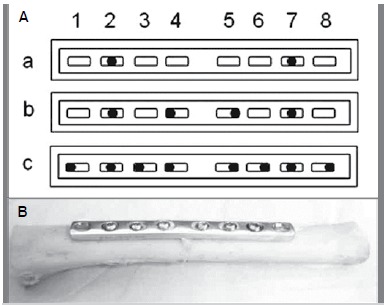
Diagram of plate fixation (A - above) and a real bone and plate assembly (B - below).

Fixation was done according to the technique recommended for the real procedure in humans, adapted to the requirements of the investigation.^18^ With the DCP plate adapted onto the dorsal aspect of the bone segment with special forceps, the screws were introduced alternately and centrifugally on each half of the plate. (Figure 2) All 16 mm-long 3.5 mm in diameter screws were inserted according to the appropriate technique, as follows: 1) drilling of a 2.5 mm wide diametrical hole through both cortices; 2) tapping the internal screw thread with a 3.5 mm in diameter tap; 3) introducing the screws with a hexagonal key; and 4) final tightening with two fingers.

Experimental Groups: Both coronal and sagittal diameters were measured at the exact midpoint of the specimens with a precision pachymeter. The sagittal diameter of the assemblies included the plate thickness. UV measurement procedures were carried out as follows for each group:

Group 1: Intact bone segments (no plate) (n=10);

Group 2: ST plate assemblies (n=10);

Group 3: DCP plate assemblies (n=10).

UV was measured in both the coronal (subgroups C) and sagittal (subgroups S) planes for all groups. Five sequential measurements were made for each specimen in each plane, with the greater and the smaller values being discarded. An average value was then calculated from the remaining three and used for interpretation and statistical analysis.

**Figure 3:**
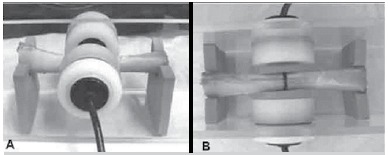
A diaphyseal bone segment positioned into the acoustic tank, between the emitting and recipient transducers.

Ultrasonometric analysis: An acoustic tank equipped with two diametrically opposed unfocused ultrasound transducers (2 mm-thick PZT-5 disc, 20 mm in diameter, 1 MHz frequency), one for emission and the other for reception, was used. The assemblies were positioned lengthwise into the tank, transversely between the transducers, with the central diameter of the specimen aligned with the longitudinal axis of both transducers. UV measurements in the coronal plane were made with the specimens positioned in such a way that the plate faced downwards, therefore out of the way of the ultrasound emission (subgroups 2C and 3C). UV measurements in the sagittal plane were made with the plate directly facing the emitting transducer, therefore interposed in the way of the emission (subgroups 2S and 3S). A 4 mm-distance was maintained between the transducers and both sides of the specimen. (Figure 3)

**Figure 4:**
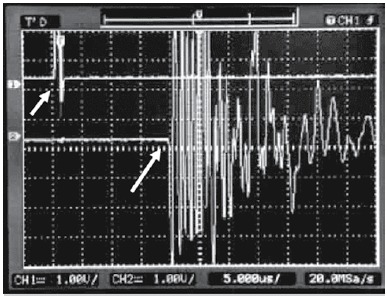
The emitted US wave (short arrow) and the first arrived US signal (long arrow), as seen on the oscilloscope screen.

Ultrasound wave pulses were produced by an ultrasound generator-receiver-amplifier source (Biotecnosis do Brasil Ltda., Model US01, Ribeirão Preto SP Brazil) able to generate high power narrow well defined ultrasonic pulses (1 MHz frequency, 1 µs pulse duration, 0.1 ns rise time, 1s repetition) and linked to a digital storage oscilloscope (Agilent Technologies, Inc., model DSO3062A, Shangai, China) and to a computer loaded with a specific software for automatic calculation of the UV. The oscilloscope permitted reckoning whether the received signal needed amplification and finding the proper portion of the ultrasound wave generated and received by the equipment. The first arriving signal (FAS), as recognized on the oscilloscope screen, was the parameter used to check the time required for the ultrasound pulse to reach the recipient transducer. (Figure 4) Time values were automatically transferred to the above mentioned software, but distance (diameter) was manually inserted for each individual specimen. UV was then automatically calculated according to the principle of the difference between the time required for the ultrasound waves to travel through the reference medium alone (water) first and then through this and the specimen.^19^^,^ 20

The calculation was performed according to the equation:



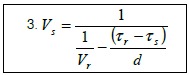



Where: *V* *_s_*: velocity through the specimen; *V* *_r_*: velocity through the reference propagation medium (water); τ*_r_*: time for reference propagation medium alone (water); τ*_s_*: time for reference propagation medium and specimen; and *d*: distance (diameter of specimen).

Before starting any measurement procedure, the system was calibrated with a compact 23 mm-thick Teflon disk of known and constant UV (1250 m/s, ±0.3%). Water temperature was maintained at 35ºC by heating to avoid variation of ultrasound speed.^21^

### Statistical analysis

The PRC GLM procedure of the SAS^(r)^ 9.0 software was used for the statistical analysis at the 1% level of significance (p<0.01). Data were first submitted to analysis of variance by the method proposed by Montgomery,^22^ according to which the total variance of a determined response (dependent variable) is divided into two parts, the first referring to the linear regression between groups, and the second referring to the residues, or errors, within groups. The larger the former in relation to the latter, the larger the difference between means of the compared groups, assuming that the residues are normally distributed, with 0 (zero) as the mean value; a logarithmic transformation was applied to the variable response whenever this assumption was not satisfied. Comparisons were made using the orthogonal contrasts, based on the Student's *t* distribution.

## RESULTS

All specimens prepared and analyzed were included in the study, none being discarded due to divergent results, meaning that all groups were quite homogenous.

The mean density of the bone segments was 1416 kg/m^3^

(range: 1219.04 - 1626.92 kg/m^3^). Acoustic impedance is 46.2 x 10^6^ kg/m^2^/s for steel (UV: 5900 m/s; density: 7830 kg/m^3^), 3.66 x 10^6^ kg/m^2^/s for bone (UV: 2587 m/s; density: ±1416 kg/cm^3^) and 1.4 x10^6^ kg/m^2^/s for water (VPUS: ±1400 m/s; density: ±1000 kg/m^3^), accounting for reflection coefficients of 0.88 for the water-steel interface, 0.72 for the steel-bone interface and 0,14 for the water-bone interface. Therefore, the percentage of the emitted ultrasound energy reflected by the interfaces is 88%, 72% and 14%, respectively, resulting in the fact that only about 12% and 28% actually go through the plate and the bone, respectively, not considering the re-reflection inside each material.

**Figure 5:**
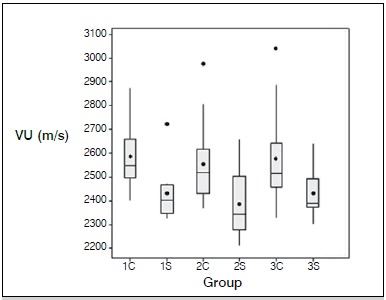
Box plot graph of the both coronal (C) and sagittal (S) UV, according to group. The sagittal UV was consistently lower than the coronal UV.

The mean transverse coronal UV was consistently higher than the mean transverse sagittal UV in all three groups. (Table 1, Figure 5) The mean transverse coronal UV was 2587.5 m/s (range: 2399 - 2876 m/s), 2555.7 m/s (range: 2365 - 2977 m/s) and 2576.8 m/s (range: 2328 - 3040 m/s), with medians of 2550, 2520 and 2516 m/s, in subgroups 1C, 2C and 3C, respectively. The mean transverse sagittal UV was 2430.8 m/s (range: 2323 - 2725 m/s), 2385.7 m/s (range: 2210 - 2657 m/s) and 2429.7 m/s (range: 2302 - 2640 m/s), with medians of 2402.5, 2342.5 and 2387.5 m/s, in subgroups 1S, 2S and 3S, respectively.

**Tabela 1:** Descriptive statistics of the coronal and saggital UV (m/s).

	**Group**	**n**	**Mean**	**SD**	**Mínimum**	**Median**	**Maximum**
Intacto	1C	10	2587.5	158.14	2399	2550	2876
	1S	10	2430.8	115.68	2323	2402.5	2725
Semitub	2C	10	2555.7	192.83	2365	2520	2977
	2S	10	2385.7	150.31	2210	2342.5	2657
DCP	3C	10	2576.8	217	2328	2516	3040
	3S	10	2429.7	96.83	2302	2387.5	2640

A significant difference (p=0.009) was only observed between subgroups 1C (coronal plane, untouched bone) and 2S (sagittal plane, semitubular plate). For the remaining comparisons, the p value ranged from 0.0132 (2S x 3C) to 0.9884 (1S x 3S). (Table 2) According to these results, the stainless steel plates did not significantly change the transverse propagation of ultrasound waves along the bone.

**Table 2:** Statistical comparison of the mean UV between groups.

**Comparison**	**Mean**	**Difference**	**P-value**	**CI (95%)**	
1C-1S	2587.5	156.7	0.0409	6.6	306.79
	2430.8				
1C-2C	2587.5	31.8	0.6748	-118.29	181.89
	2555.7				
1C-2S	2587.5	201.8	0.009	51.7	351.89
	2385.7				
1C-3C	2587.5	10.7	0.8877	-139.39	160.79
	2576.8				
1C-3S	2587.5	157.8	0.396	7.7	307.89
	2429.7				
1S-3C	2430.8	-146	0.0564	-296.09	4.09
	2576.8				
1S-3S	2430.8	1.1	0.9884	-148.99	151.19
	2429.7				
2C-2S	2555.7	170	0.269	19.9	320.09
	2385.7				
2C-3C	2555.7	-21.1	0.7807	-171.19	128.99
	2576.8				
2C-3S	2555.7	126	0.0988	-24.09	276.09
	2429.7				
2S-3C	2385.7	-191.1	0.132	-341.19	-41
	2576.8				
2S-3S	2385.7	-44	0.5618	-194.09	106.09
	2429.7				
3C-3S	2576.8	147.1	0.0546	-2.99	297.19
	2429.7				

CI: Confidence interval.

## DISCUSSION

Ultrasonometry basically consists of the measurement of the propagation velocity and attenuation of the ultrasound energy through the bone, where it can be influenced by anatomical accidents, such as a fracture. The effect of such accidents is the reduction of the propagation velocity as measured with specific equipment, so that periodical evaluations permit establishing comparisons and can help predict whether a fracture is healing or not. In fact, the results of many investigations demonstrate that transmission ultrasonometry can precisely indicate the healing status of a fracture, therefore introducing a new diagnostic method with great potential for clinical application, with the advantage that it does not involve ionizing radiation.^23^^,^ 24

The healing process of a fracture implies quick and drastic changes in the consistency of the tissue around the broken bone and is considered complete by about the sixth month, after a great deal of remodeling has taken place, thus restoring the normal microscopic structure of the bone. The macroscopic appearance of the bone can also be restored and this can be easily seen on conventional radiographs, particularly for conservatively treated fractures. However, a considerable number of fractures do not heal and evolve to a healing anomaly (delayed union, nonunion), thus requiring specific treatment the efficacy of which largely depends on the time of recognition of the problem. The sooner the diagnosis is firmed the better, the ideal time being around the third month, a period during which an uneventful healing should already have occurred. For conservatively treated fractures, the diagnosis of a healing anomaly is not particularly difficult, based on the clinical detection of abnormal mobility and pain at the fracture site and on the usually typical radiographic findings. At the present time, the diagnostic problems of a healing anomaly most often involve fractures surgically treated with the use of metal implants, for two main reasons: first, the fixation can still be stable, therefore preventing the detection of abnormal mobility, and second, the metal implants such as plates and screws or intramedullary rods prevent the

thorough visualization of the fracture itself and of the healing status. As a consequence, a healing anomaly can go undiagnosed within the ideal period (three months for delayed union, six months for nonunion), with serious economic and social loss for the patient, whose recovery is delayed.

The surgical treatment of fractures has been growing in geometrical proportions, particularly in large medical centers, since the introduction of modern fixation techniques and more reliable metal implants beginning some thirty years ago. Actually, it is not possible to overlook the fact that the internal fixation of fractures is quickly becoming the gold standard for the treatment of virtually all types of fractures, particularly those of the shaft of long bone in adults. There are no specific general statistics on the incidence of complications of the surgical treatment of fractures (infection, loosening, delayed union and nonunion, among others), but it is a well known fact that they increase in the same proportion as the operations, including the healing anomalies. It is probably here that ultrasonometry will find its best clinical application.^25^

However, in contrast to the greater tendency to operate fractures, most of the investigations on the use of ultrasonometry to diagnose the healing status were addressed to conservatively treated fractures.^4^^,^ 11 ^,^ 26 ^,^ 27 It was, therefore, the objective of the present investigation to study the influence of osteosynthesis plates on the UV through the bone. Instead of a fracture or osteotomy, an intact bone was used in order not to introduce other variables in the investigation, perhaps of difficult interpretation at this stage. Then, diaphyseal segments of fresh frozen sheep tibiae were used in combination with two types of stainless steel plates, not coincidentally the ones most used for the fixation of fractures small long bones (radius, ulna, fibula, and clavicle), exactly to mimic the fixation procedure of this type of bone. Both were 8-hole 3.5 mm plates, compatible with the dimensions of the bone segments used, and were fixed rigorously according to the recommended technique for a real surgical procedure. Underwater ultrasonometric analyses were undertaken, the water acting like the soft tissues around the bone, since this is the modality capable of transmitting most of the ultrasonic energy from the emitting to the recipient transducer, passing through the bone-plate assemblies with at least three interfaces. Also, instead of the axial modality, the transverse modality was preferred for this investigation, since it seems to be the most suitable for clinical application, particularly for the deep long bones enveloped by a thick muscular layer.

Ultrasound waves are generated by an unfocused emitting transducer as a cone-shaped emission, but over 80% of the waves concentrate in a central portion called Fresnel zone, of about the same diameter as the transducer itself and within which the waves are practically parallel to each other.^28^^,^ 29 In the present case, transducers measuring 20 mm in diameter were used, purposefully wider than the bones (±15 mm in diameter) and assemblies, to compensate for the addition of 0.8 mm for the semitubular plate and 3 mm for the DCP plate, which resulted in about 16 mm in diameter for the former and 19 mm for the latter, on the sagittal plane. The mean UV for the intact bones observed here (2587 m/s on average) was somewhat lower than that observed in our two previous investigations (mean 2890 m/s and 2940 m/s on average, respectively), in which the transducers used were 12 mm in diameter. The signal referring to the steel plate was always the first to arrive, as a small positive indentation with very small amplitude, followed by the signal referring to the bone, a few times wider. The ultrasound waves at the periphery of the cone-shaped emission reflected and re-reflected on the acoustic tank walls until they completely dissipated in the water. This was a similar phenomenon for all specimens and roughly corresponded to the clinical situations in which the exact diameter of the tested bone is not precisely known. It also represents valid information for both manufacturers of ultrasound equipment and practitioners dealing with the problem of adjusting the diameter of the transducers to that of the bone.

The ultrasound wave propagation pattern is different for bone and for steel, although both are anisotropic elastic materials, because they have an entirely different internal structure, the result of which is the great difference in UV. Also, for a normal (90º) incidence emission as used here the ultrasound waves propagate superficially, if the wavelength is smaller than the cortex thickness, therefore only supplying information about the periosteal region; on the contrary, for a wavelength greater than the cortex thickness, the waves propagate through the entire cortex thickness, therefore providing more complete information about the whole bone structure.^30^ The 1 MHz frequency emission used here produced ultrasound waves of 1.5 mm wavelength, which is about the thickness of the cortex of the analyzed bones, therefore indicating that the ultrasound propagation followed the pattern described above.

Furthermore, the cylindrical shape of the bone shaft imposes a ring-fashion pattern to the wave propagation, the waves running along both anterior and posterior cortices to exit on the opposite side of the emission together with a smaller amount of waves which manage to go directly through the bone marrow, perhaps with a lower UV.^31^ In this case, the measured UV would be then an average of both cortical and marrow velocities. The stainless steel plate was intimately united to the bone, the two acting as a single piece for both coronal and sagittal examinations, with the wave propagation probably following the same pattern for the bone segments alone.

Because of the much higher UV for the steel than for the bone, before starting the investigation we thought that the combination of the steel plate with the bone would result in higher UV for the assemblies than for the bone alone. However, our results showed that the steel plate actually caused the UV to decrease in the assemblies, particularly on the sagittal examination, although not significantly. We suspect that the higher reflection coefficient of steel may have played a role in this case, since less than 12% of the ultrasound energy can manage to go through the water-steel interface, the plate acting as a shield to reflect most of the ultrasound energy. The steel-bone interface may also contribute to reducing UV, since only 28% of the ultrasound energy can go through it. The result of the action of the two reflecting interfaces for the sagittal examination is that only about 4% of the emitted energy actually crosses the assemblies and hits the recipient transducer, with most of it being lost due to reflection, reason why the FAS may need amplification to be perfectly recognized. For the coronal examination, without the plate in the way, total reflection in the water-bone interface is much smaller (14%), about 86% of the ultrasound energy managing to propagate through the bone, to be hindered only by the complex inner structure of the bone alone, with two cortices and the bone marrow, thus resulting in a more easily recognized FAS. Nevertheless, sagittal UV was not significantly different from coronal UV, although being slightly lower.

## CONCLUSION

We conclude that the addition of the two types of stainless steel osteosynthesis plates does not significantly change UV through the bone, therefore not preventing the use of ultrasonometry to study the healing status of fractures treated by operative means with the use of metal implants.
